# Methodological Approaches to Support Process Improvement in Emergency Departments: A Systematic Review

**DOI:** 10.3390/ijerph17082664

**Published:** 2020-04-13

**Authors:** Miguel Angel Ortíz-Barrios, Juan-José Alfaro-Saíz

**Affiliations:** 1 Department of Industrial Management, Agroindustry and Operations, Universidad de la Costa CUC, Barranquilla 081001, Colombia; 2 Research Centre on Production Management and Engineering, Universitat Politècnica de València, 46022 Valencia, Spain; jalfaro@omp.upv.es

**Keywords:** healthcare, emergency department, process improvement, systematic review

## Abstract

The most commonly used techniques for addressing each Emergency Department (ED) problem (overcrowding, prolonged waiting time, extended length of stay, excessive patient flow time, and high left-without-being-seen (LWBS) rates) were specified to provide healthcare managers and researchers with a useful framework for effectively solving these operational deficiencies. Finally, we identified the existing research tendencies and highlighted opportunities for future work. We implemented the Preferred Reporting Items for Systematic Reviews and Meta-Analyses (PRISMA) methodology to undertake a review including scholarly articles published between April 1993 and October 2019. The selected papers were categorized considering the leading ED problems and publication year. Two hundred and three (203) papers distributed in 120 journals were found to meet the inclusion criteria. Furthermore, computer simulation and lean manufacturing were concluded to be the most prominent approaches for addressing the leading operational problems in EDs. In future interventions, ED administrators and researchers are widely advised to combine Operations Research (OR) methods, quality-based techniques, and data-driven approaches for upgrading the performance of EDs. On a different tack, more interventions are required for tackling overcrowding and high left-without-being-seen rates.

## 1. Introduction

Emergency departments (EDs) are perceived as 24/7 portals where a rapid and efficient diagnosis, urgent attention, primary care, and inpatient admission is provided for stabilizing seriously ill and wounded patients, including those with life-threatening conditions ranging from different head injuries to heart failures. EDs have assumed a wider role in the integrated healthcare system and are therefore cataloged as the cornerstone of the safety net. Furthermore, EDs play a key social role by offering access to the healthcare system for both insured and uninsured patients. Their importance in the healthcare system is also underpinned by the fact that more than half of the hospital activity takes place in their settings. Besides, as a “care hub”, it is a point of interaction between communities and hospitals.

Nonetheless, several serious problems have become glaring in EDs, even in developed countries, and must be therefore thoroughly addressed to ensure low early mortality rates and complications, increased patient satisfaction, timely emergency care, and long-term morbidity. Not surprisingly, these growing deficiencies greatly contribute to the acceleration of healthcare costs which increases the financial pressures on hospitals and shrinks their profits. The problem is even more critical as demands on ED services are expected to continue to steadily and dramatically rise in the near future which will end up amplifying the negative effects here described, while keeping EDs under a constant strain [1]. There is then an urgent need for aggressive improvements through the efficient use of inpatient resources and the implementation of operational changes in the healthcare delivery.

From this perspective, it is essential to count on the support of suitable methodological approaches to assist decision makers along the emergency care journey. The novelty of the study then lies on the need of providing orientation as well as a scientific evidence base to healthcare administrators, clinicians, researchers, and practitioners on what process-improvement methodologies can be used to fully understand and tackle the top-five leading problems presented in EDs [2,3]: Overcrowding, prolonged waiting time, extended length of stay (LOS), excessive patient flow time, and patients who leave without being seen (LWBS). Previous reviews have been conducted relating to this topic; some of them focused on critically reviewing the implementation of specific approaches to address different ED problems. For instance, some authors analyzed the use of lean thinking and its effects on ED processes [4,5,6], while others studied the contribution of discrete-event simulation implementations to tackle overcrowding and model the ED performance [7,8,9]. Saghafian et al. [10] have also discussed the contribution of operations research/management methods to the optimization of patient flow within EDs. Other works directly concentrated on assessing the effectiveness of interventions to reduce the number of frequent users of EDs [1,11], minimize ED utilization [12], decrease overcrowding [13,14], diminish the number of non-urgent visits [15], shorten the total flow time (TFT) [16] and reduce the number of patients who leave the ED without being seen [17]. Despite the considerable effort made in these studies, the review of the evidence base is still scant and narrow since: (i) the above-cited reviews are mostly focused on a particular ED problem, (ii) the aforementioned works are predominantly skewed to the use of a specific technique or approach in the ED context; therefore, there are no studies considering the wide variety of process-improvement methodologies that can be applied for the solution of the leading ED deficiencies (overcrowding, prolonged waiting time, extended length of stay, excessive patient flow time, and patients who leave without being seen - LWBS), and (iii) the use of hybrid methods has not been incorporated in the aforementioned works, thereby greatly restricting their application in the wild and the subsequent achievement of better operational outcomes. This paper hence addresses these gaps in knowledge through a systematic review focused on establishing the most popular process-improvement approaches that have been used for tackling each of the five-top leading problems in EDs. Thereby, our article lays the groundwork for analyzing the continuing evolution of this research field, devising and implementing cost-effective solutions to the leading ED problems, detecting the limitations in current practice, and identifying promising opportunities for future investigation.

**Figure 1:**
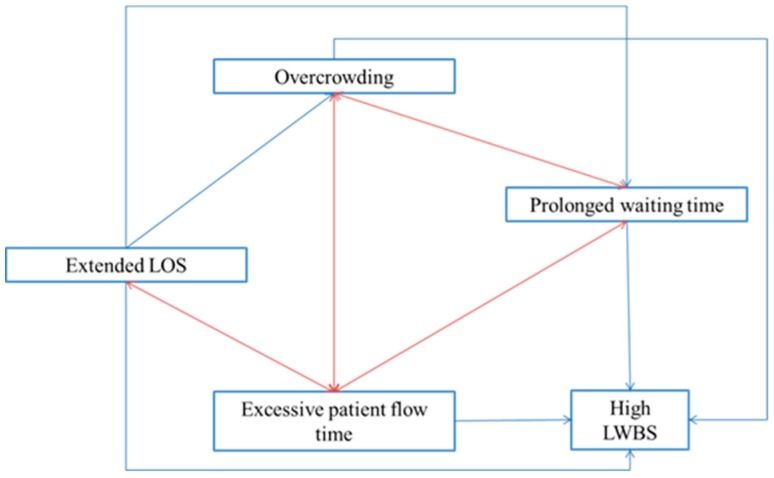
Impact-digraph map for interrelations among leading problems in EDs.

Although more deficiencies have been addressed and reported throughout the literature, we particularly focused on the above-mentioned problems due to their big impact on financial sustainability and emergency care delivery. Indeed, these problems are interconnected in several ways along the ED patient journey as described in Figure 1 (where the red and blue arrows represent feedback and dependence interrelations, respectively). On one hand, crowded emergency departments hamper the delivery of timely care which ends up increasing the total flow time within the ED setting. Indeed, some patients decide to leave the ED without being seen when these units experience long overcrowding episodes. The LWBS rates are also correlated to excessive patient flow time, long waits in the ER, and extended LOS as also pointed out in [17]. In the meantime, long stays in ED settings break the balance between demand and ED capacity which leads to overcrowding, long queuing time, and non-optimal patient journey. The aforementioned statements are evidence of strong interrelations among the foremost leading problems in EDs which is often found in healthcare environments [17]. It can be therefore inferred that improvement initiatives on some of these elements may cause a positive effect on the entire emergency delivery system by contributing to the solution of highly correlated problems. Our study will delve into these deficiencies for better understanding on their causes and consequences while identifying the methodological approaches used for their solution.

### 1.1. The Top-Five Leading Problems in EDs: Causes and Consequences

#### 1.1.1. Overcrowding

Overcrowding in EDs is the result of the imbalance between the demand for emergency care and their physical or staffing capacity. Overcrowding has become a global serious concern and continues to cause excessive waiting time, poor clinical results, patient dissatisfaction, aggressive behavior and augmented suffering for patients on pain [16]. In some cases, this problem has reached desperate proportions and crisis levels [18]. After critical analysis, it was found that this phenomenon is caused by a set of mismatches along the supply chain within the healthcare systems [19]. Some mismatches are inpatient bed availability, demand growth, and the increased proportion of non-urgent visits. It is then urgent to devise a variety of initiatives for alleviating this problem and minimizing the aforementioned negative effects on patients.

#### 1.1.2. Prolonged Waiting Time

Waiting time (WT) is defined as the interval between patient arrival and the first contact with a doctor. This is a common measure in EDs which are interested in delivering timely medical care. In addition, multiple studies have concluded that timeliness is an essential contributor to patient satisfaction with EDs [20,21]. In fact, prolonged waiting times result in patient dissatisfaction, delayed admission of new patients, more severe complications and increased morbidity. In this regard, WTs are considered as barriers to access to healthcare which is one of the primary concerns of governments and control agencies. As noted above, long waits for care are dangerous for patients, it is thus necessary to examine the determinants responsible for this problem and attempts to tackle it by implementing effective initiatives that better comply with government healthcare standards.

#### 1.1.3. Extended Length of Stay (LOS)

Emergency department length of stay (ED-LOS) is described as the time elapsed from a patient is admitted to the ED until the patient is physically discharged from this unit [22]. An extended ED-LOS may cause bypass, critical-care divert status, increased inpatient costs, higher risk of adverse events and low patient satisfaction. ED-LOS is also an important indicator of crowding and provides a decision-making basis for performance and efficiency improvement. Delays in delivery of lab and/or radiology test results, lack of hospital beds, hospital transfers taking a long time, insufficient medical staff during peak hours and other factors have been found to explain ED-LOS variation [22]. To face this problem, health authorities have incorporated policies to decrease ED-LOS as outlined with the 4-hour target in the UK [23]. Some of them have led to fewer extended LOS within the ED. However, it is still necessary to deploy interventions along the entire ED patient journey with a special focus on each component of the acute care chain.

#### 1.1.4. Excessive Patient Flow time

Patient flow is critical for delivering high quality care to patients admitted within EDs. Being aware of its importance; ED managers should continuously tackle the factors hampering the emergency care provided along the patient journey. Major causes contributing to prolonged flow time include departmental layout, insufficient medical staff, and inefficiencies of parallel assisting processes. Also, mismatches between the demand on emergency services and ED capacity have been associated to this problem [2]. If improved, elevated patient satisfaction rates and reductions in mortality and morbidity can be expected in conjunction with a significant lessening of the consequent financial burden assumed by healthcare systems. However, as patient journey is affected by intrinsic factors and multiple interactions with other services, more robust and advanced methodological approaches are required for assisting decision-makers in designing cost-effective interventions considering both the complexity of emergency care systems and the expected increased demand.

#### 1.1.5. High Number of Patients Who Leave the ED without Being Seen

Patients who leave without being seen (LWBS) are more prone to experience worsening health compared to those who were attended. Additionally, LWBS are more likely to be readmitted within the next few hours with more severe complications which results in the use of more complex services and increased healthcare costs. The rate of LWBS is then considered as a quality metric of concern in healthcare systems [17]. Meanwhile, restricted ED capacity, long WT for triage classification, and diversion status are among the most common causes of this problem. It is therefore important to ensure a correct provision of ED services by developing effective initiatives that consider the above-mentioned factors and their interactions.

## 2. Methods

### 2.1. Framework for Literature Review

**Figure 2:**
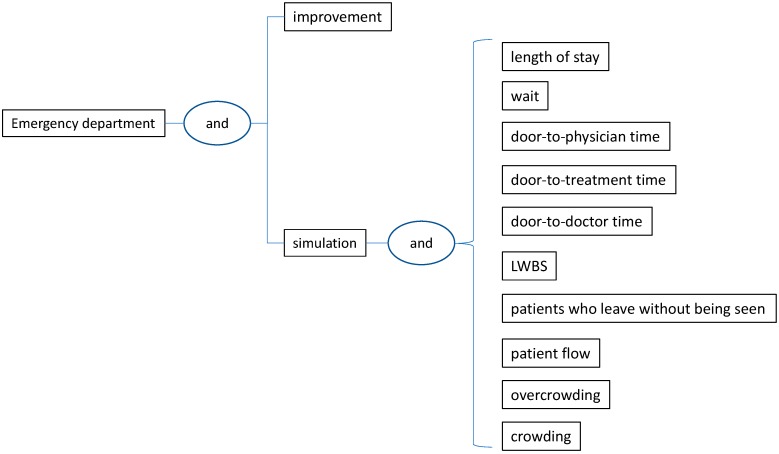
Search algorithms used in the literature review.

This review aims at identifying research papers published in high-quality journals and focused on interventions addressing the above-mentioned leading problems in EDs. A paper is considered in this review if it evidences and discusses the implementation of methodological approaches for process improvement in EDs. The articles also had to be written in English and present data supporting the results obtained from the application. Research articles presenting conceptual models without validation in the wild were discarded from this study. Moreover, conference papers, doctoral dissertations, textbooks, master’s thesis, and review papers were excluded from this study. Based on this perspective, we followed the Preferred Reporting Items for Systematic Reviews and Meta-Analysis (PRISMA). PRISMA guidelines help to report systematic reviews, especially appraisal of interventions as aimed in this study. By using different search algorithms (Figure 2) in a set of high-quality databases, we covered an extensive range of methodological approaches that have been implemented for the solution of the leading ED problems. Initially, we conducted an extensive review of the international literature published from April 1993 (the date in which the first paper was published) until October 2019, in multiple databases including ISI Web of Science, Scopus, PubMed, IEEE, Google Scholar, ACM Digital Library and Science Direct. The search algorithms used in this review are presented in Figure 2. Such algorithms include the most popular improvement techniques and the top-five leading problems in EDs. In particular, techniques like “simulation”, “lean”, “six sigma”, “queuing”, “critical pathways”, “continuous quality improvement”, “regression”, “decision-making”, “integer programming”, “linear programming”, “optimization”, “game theory”, and “markov” were considered in these algorithms. Although our coverage is limited to approaches from the industrial engineering domain, other strategies including clinical-related interventions, personnel training, the ABCDE of Emergency care, and Triage can be also implemented for minimizing the impact of the leading ED problems.

**Figure 3:**
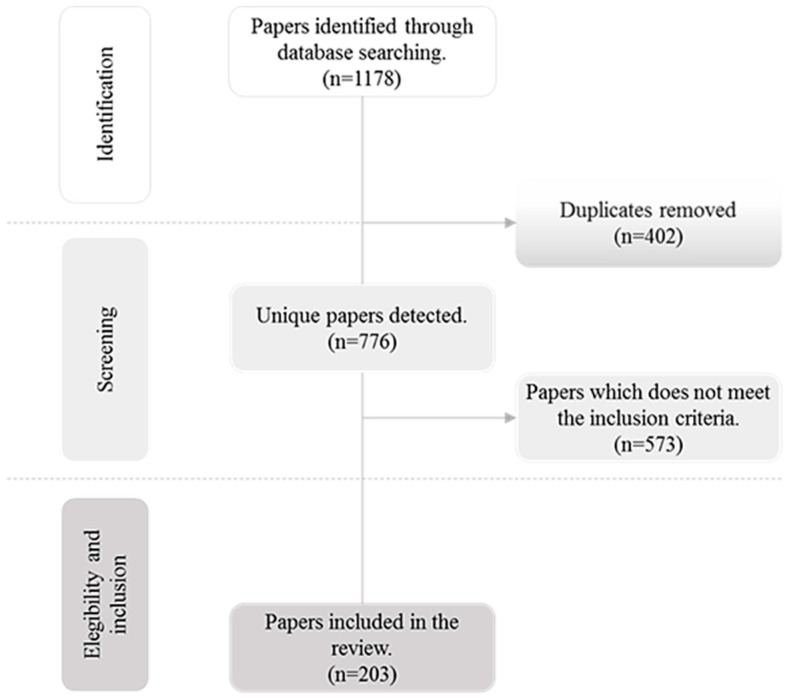
PRISMA flow diagram.

Figure 3 shows the PRISMA flow diagram describing the review process. Two independent reviewers studied the paper abstracts returned by the search engines for first screening. After initial selection, both reviewers thoroughly revised the papers to determine whether they met the aforementioned inclusion criteria. The articles satisfying these conditions were thoroughly examined in full size for a deeper understanding of the methodological approach. The papers were then independently extracted and classified according to the targeted ED problem (overcrowding, prolonged waiting time, extended length of stay, excessive patient flow time, and patients who leave without being seen - LWBS). In this classification scheme, we also pointed out the techniques that have been used for tackling each of these deficiencies so that healthcare managers, researchers, and practitioners can effectively implement them in the wild. The articles were further categorized and analysed considering the publication time. After applying this review scheme, we narrowed the initial list of papers (*n* = 1178) to 203 distributed in 120 journals. The classification results are presented in the next section.

### 2.2. The Process-Improvement Methodologies Used for Tackling the 5-Top Leading Problems in EDs

The increasing concern of policy makers, ED managers, practitioners, and researchers for constantly improving the emergency care delivered to patients while reducing cost overruns is the main motivation for classifying the selected papers according to the targeted ED problem. In this scheme, 203 papers were categorized as follows: (1) Extended length of stay (LOS) (2) Prolonged waiting time (3) Excessive patient flow time in ED (4) Overcrowding, and (5) High number of patients who leave without being seen (LWBS). Table 1 summarizes the number and percentage of selected papers contributing to the solution of each problem. Table 1 also presents useful information in reference to the annual frequency of publication. Then, Table 2, Table 3, Table 4, Table 5 and Table 6 list the articles per each of the 5-top leading problems in conjunction with the related process-improvement techniques. These tables also specify whether the studies have used either a single or hybrid approach for solving the related ED problem. Further comments are made on these studies for identifying useful insights that can be considered for implementations in the real ED context. Additionally, the most popular techniques solving each ED problem are identified and discussed on the use of single/hybrid approaches. Thereby, we provide decision-makers with a robust methodological framework underpinning the design of cost-effective solutions.

According to Table 1, the ED problems with the highest number of papers evidencing the use of process improvement methodologies were (Table 1): “Extended length of stay” (53.20%; *n* = 108 papers) and “Prolonged waiting time” (46.79%; *n* = 95 papers). On a different tack, only 25 papers (12.31%) were related to targeting a reduced LWBS which proves that this research field as at the earlier stages. Further details on these papers are commented below for deeper understanding and analysis.

## 3. Results

Identifying the process-improvement approaches that have been implemented for addressing the top-five leading problems is critical for guiding healthcare managers, decision-makers, researchers, and other stakeholders towards the design of effective interventions improving the emergency care provided to patients while shortening the operational costs. For this purpose, the following sub-sections will focus on pointing out the most prominent techniques, either single of hybrid, in each ED problem whereas highlighting the main advantages justifying their use in the practical clinical scenario.

### 3.1. Papers Focusing on Reducing the Extended LOS

Table 2 lists all the contributions targeting a reduced LOS within EDs. According to the reported literature, this is the ED problem with major interest among researchers and practitioners. This is since extended LOS has become an international threat to public health considering its significant association with decreased disaster response, cost overruns, patient dissatisfaction, and poor clinical outcomes including increased mortality rates [24]. In an effort to address this problem, several studies have presented different process improvement approaches with implementation in the real ED context. Based on the review, 66.66% (n = 72 papers) of the papers evidenced the use of a single approach whilst 33.34% (n = 36 papers) tackled the extended LOS using a combination of two or more techniques. In particular, 63.88% (n = 23 papers) out of the hybrid-approached papers employed two methods, 30.55% (n = 11 papers) integrated three techniques, and 5.55% (n = 2 papers) mixed four methods as evidenced in Easter et al. [25] and Fuentes et al. [26].

Different process improvement methods have been combined for better assisting ED managers in addressing the prolonged stays in EDs. The first hybrid-approached contribution was produced by Ross et al. [117] who mixed continuous quality improvement with critical pathways to diminish the LOS at the emergency department of Macomb Hospital Center (Warren, MI, USA). Thanks to this approach, LOS decreased from 7.52 days to 6.33 days for stroke patients. Other studies have combined simulation with other operations research (OR) methods. For instance, Ashour and Kremer [129] integrated simulation with Multi-attribute Utility Theory (MAUT) and Fuzzy Analytic Hierarchy Process (FAHP) for developing a triage algorithm that classifies emergency patients. The simulation evidenced that MAUT-FAHP outperforms the Emergency Severity Index for ESI levels 2–5 with a significant reduction of ED-LOS. Another related work is presented by Bish et al. [99] who merged simulation with queuing analysis for shortening the median LOS in an adult ED located in New Jersey. In this case, the results evidenced that this measure was shortened from 192 to 112 min. Other studies combining simulation and queuing theory can be found in Ferrand et al. [105] and Zeltyn et al. [128]. Another related study was presented by Chen and Wang [102] who proposed an integrated approach integrating non-dominated sorting particle swarm optimization (NSPSO), multi-objective computing budget allocation (MOCBA) and discrete-event Simulation (DES) aiming at meeting the government LOS targets in Sunnybrook Hospital emergency department.

The combination between simulation and design of experiments (DOE) has been also employed for the scientific community and decision-makers when targeting shortened LOS. An interesting related intervention is exposed by Kaner et al. [111] who used this approach for formulating improvement scenarios with data derived from a real-life ED environment. Such framework is called to replace the well-known trial-and-error experiments often used when pretesting interventions on ED-LOS. Other works implementing the simulation-DOE approach are described in Aroua and Abdulnour [130] and Visintin et al. [124]. Integrating simulation and lean techniques is another alternative adopted by researchers and practitioners when dealing with excessive stays in EDs. For example, Romano et al. [116] used this approach in conjunction with causal loop diagrams for minimizing the LOS and waiting times in Italian hospitals. Specifically, a new ED configuration was pretested considering the partial reassignment of unused beds and medical staff to patients with white code only. Another research using this integration is presented by Lo et al. [113] who implemented an electronic provider documentation (EPD) in a pediatric ED. In this case, simulation allowed testing potential affectations on ED-LOS when transitioning from paper charting to EPD. Other integrated methodologies including simulation are reported by Abo-Hamad and Arisha [131], Ashour and Kremer [20], Easter et al. [25], Yousefi et al. [127], and Yousefi and Ferreira [125]; however, their application has not been replicated throughout the literature.

Also, hybrid approaches excluding simulation techniques were considered to address the prolonged ED-LOS. Some of them are a mix of OR approaches as noted in He et al. [109] and Sir et al. [122]. Other papers combine different statistical techniques as evidenced in Fuentes et al. [26], Huang et al. [110], Techar et al. [123], and Ross et al. [118]. Another category includes the mix of lean manufacturing and other techniques as exposed in Blick [100], Chadha et al. [101], and Furterer [106]. LM encompasses a wide variety of process-improvement techniques focusing on eliminating wastes detected in the value chain of ED processes. Besides, it provides a comprehensive way of shortening buffering costs, increasing process efficiency, and fostering CQI culture. Likewise, it has become a good alternative for delivering the upmost value to ED patients by delivering effective care.

As presented above, single methods have been widely used by decision-makers and researchers when targeting shortened stays in EDs. Some studies have addressed this problem through a quality improvement technique (i.e., lean manufacturing, continuous quality improvement). One of the most popular approaches in this domain is lean manufacturing (LM, 20 papers = 27.77%). In this regard, Allaudeen et al. [48] performed a multidisciplinary lean intervention where root causes of delays were properly identified and tackled. In fact, the ED LOS for medicine admissions decreased by 26.4% from 8.7 to 6.4 h (*p*-value < 0.01). Another application is presented by Carter et al. [50] who applied LM techniques for improving the clinical operations of an ED located in Ghana. Their article provides important lessons to be considered during the implementation of LM in the ED context.

The second most used method from quality domain was continuous quality improvement (CQI) (*n* = 7 papers = 9.72%). The most recent work employing QI is cited in Lovett et al. [80] who reported an intervention at a multi-campus academic health system where immediate improvements were enhanced in relation to LOS. Other works employing QI can be seen in Brent et al. [76], Fernandes and Christenson [77], Fernandes et al. [78], Higgins III and Becker [79], Preyde et al. [81], and Rehmani and Amatullah [82]. The application of six sigma [93], PDSA cycle [84,85], and ED dashboard/reporting application [98] were also detected in the literature as part of the multiple quality-based methods that have been applied for solving the excessive LOS problem in EDs.

Simulation was also employed in a single way to address the prolonged stays in emergency departments. Indeed, its use was reported in 29.16% (*n* = 21 papers) of the studies using single methods. One of the simulation-related interventions is observed in Gul and Guneri [31] who applied this method in an attempt to the patient average LOS in an ED of a regional university hospital in Turkey. In consequence, LOS was shortened with an improvement rate of 30%. A more recent work is exposed by Keyloun et al. [34] who modeled the implementation of a new treatment pathway taking advantage of long-acting antibiotics (LAs) aiming at estimating its effects on patient throughput rate, LOS, and cost. The outcomes evidenced a 68% reduction in patient LOS; in other words, 7.2 h less compared to the initial performance.

There is also an interest from research community in applying statistical techniques for reducing prolonged stays in emergency care settings. The reported literature revealed that 12.5% (*n* = 9 papers) of the papers using single approaches, incorporate the application of these methods when addressing the extended LOS problem. In this respect, Kaushik et al. [70] used multivariate regression analysis for identifying how a 1-minute decrease in laboratory turnaround time is associated with the emergency room LOS. In addition, Maniaci et al. [71] used linear regression based on the log of LOS for pinpointing factors associated with excessive stays in EDs. In this case, median ED LOS was found to be associated with blood alcohol concentration, urine drug test (UDT), and UDT positive for barbiturates.

OR methods were also applied in a single form for dealing with the extended LOS within EDs. For example, Ajmi et al. [83] developed an agent-based dynamic optimization model for improving several performance indicators (LOS, remaining patient care load, and cumulative waiting time) in EDs. OR-based studies addressing long LOS are evidenced in Chan et al. [91], Derni et al. [87], Liu et al. [95], and Oueida et al. [86,96]. Apart from the aforementioned single techniques, less popular methods like critical pathways [88,89,97], pivot nursing [92], and process redesign [94] were also used by some practitioners and researchers to diminish the total burden produced by long ED-LOS.

### 3.2. Papers Focusing on Reducing the Waiting Time

Table 3 presents all the papers aiming at shortening the door-to-physician time in EDs. Based on the scanned literature, this is the second most popular ED deficiency addressed by decision-makers and researchers. Prolonged waiting time has been considered as major problem within EDs given its significant association with patient dissatisfaction, increased number of complaints, and poor outcomes for patients (increased morbidity and mortality). Nonetheless, shortening waiting times at the ED is pretty challenging since it encompasses diagnosis, prioritization of patients, monitoring and management of waiting times, and provision of suitable resources. In an attempt to solve this problem, various authors have exposed different process improvement methodologies with validation in the real-world. In this respect, 55.78% (*n* = 53 articles) of the contributing works used a single method while 44.22% (*n* = 42 articles) dealt with the waiting time problem by applying an integration of two or more techniques. Explicitly, 64.28% (*n* = 27 articles) out of the hybrid-approached articles implemented 2 methods, 26.19% (*n* = 11 articles) mixed three techniques, and 9.52% (*n* = 4 articles) merged four methods as exposed in Acuna et al. [132], Ala and Chen [133], Easter et al. [25] and Yousefi and Yousefi [134].

As evidenced in the aforementioned statistics, the use of hybrid approaches has received increasing attention from decision-makers and the scientific community when targeting reduced door-to-treatment times in emergency departments. The first contribution employing this methodological framework was provided by Benson and Harp [167] who merged DES and system thinking for reducing ED waiting times. After several simulations of different improvement scenarios, the ED managers decided to reorganize the patient flow and automat hospital-wide bed control. Thanks to these interventions, door-to-doctor times were slackened by 19% in parallel to increases in patient satisfaction rates. The evidence base also reveals that 66.66% (*n* = 28 articles) out of the integrated-approached studies have adopted this technique as part of their methodological framework. Merging simulation with other OR methods has been a popular alternative for addressing the waiting time problem. For example, Zeinali et al. [184] combined metamodel techniques and simulation for minimizing the total average waiting time of an Iranian ED considering capacity and budget constraints. After intervention, the total waiting time of ED patients was reduced by approximately 48%. A similar research was presented by Kuo [174] used a simulation-optimization algorithm to support the waiting time improvement in an ED located in Hong Kong. The results revealed that the implementation of staggered shifts is helpful to decrease this metric.

The OR technique that has been mostly mixed with simulation is Queuing theory. In this regard, Izady and Worthington [173] applied discrete-event simulation, queuing models, and a heuristic staffing algorithm in a real emergency care setting for meeting the target established by the UK government (98% of the patients to be discharged, transferred, or admitted to emergency care within 4 h of arrival) and consequently applying for incentive schemes. In this case, it was concluded that meaningful improvement on the target can be gained, even without augmenting total medical staff hours. A second study utilizing this combination was performed by Xu and Chan [183]. These authors demonstrated that, based on this predictive approach, decision-makers can identify when congestion is going to increase, thus facilitating a rapid intervention on patient flow for ensuring reduced waiting times. Such an approach was proved to outperform the current policies due to its ability of reducing lengthy waiting times by up to 15%. Interesting interventions employing this integration can be also evidenced in Bish et al. [99], Ferrand et al. [105], and Zeltyn et al. [128]. Other papers integrating OR methods and simulation can be found in Ala and Chen [133], Diefenbach and Kozan [169], El-Rifai et al. [170], Ghanes et al. [107], Goienetxea Uriarte et al. [108], Oueida et al. [114], Sinreich et al. [121], and Yousefi and Yousefi [134].

Over the recent years, the use of computer simulation and DOE also set out to receive attention from practitioners related to emergency care field. For instance, Aroua and Abdulnour [130] mixed these methods for improving patient LOS of a university emergency hospital. Specifically, DOE underpinned the evaluation of improvement scenarios based on LOS variations. Other contributions employing this hybrid approach are available in Visintin et al. [124] and Zhao et al. [164]. Meanwhile, the use of DES-lean methodology is beginning to become prominent when addressing patient waiting time within EDs. Bal et al. [166] provide a walk-through of how computer simulation and lean manufacturing can be utilized for tackling the waiting time problem. In this paper, the very-well known “Value Stream Mapping” was found to be useful for detecting non-value added times within Sadi Konuk hospital ED. Similar implementations can be also found in studies such as Martínez et al. [176] and Romano et al. [116]. As a step towards reducing lengthy waiting times, other methods have been integrated with simulation: BSC and PRIME [131], DEA [162], DGP [20], statistical methods [25], machine learning [171] and group decision-making [125]. This demonstrates the flexibility and adaptability of this tool in hybridized methodologies.

Mixing OR methods, excluding simulation, has also become a popular approach among researchers and practitioners with major interest in diminishing ED waiting times. In one case, mixed integer linear programming and genetic algorithm (GA) were coupled for minimizing the total waiting time of patients in the emergency department laboratories. The proposed combination was proved to significantly reduce the total waiting time of prioritized patients [165]. More recently, Acuna et al. [132] opted to use a robust approach integrated by mixed integer programming, game theory, and single/bi-objective optimization models for improving ambulance allocation and consequently reducing patients’ waiting time in 11 EDs located in Florida. Other examples in the application of integrated OR methods when dealing with lengthy ED waits are provided in Daldoul et al. [168], He et al. [109], Lau et al. [175], Othman et al. [178], Sir et al. [122], and Umble and Umble [182]. Other combinations aiming at facing the extended waiting times are simplified in Mazzocato et al. [177], Othman and Hammadi [179], Perry [180], and Stephens and Broome [181].

Overall, single methods are also common for supporting improvement strategies targeting decreased door-to-doctor times. Undoubtedly, simulation has provided good support for reducing door-to-physician times in EDs even when used in a single way (*n* = 17 papers; 32.07% of single-approached contributions). Coughlan et al. [30] developed a simulation model to cope with the lengthy door-to-treatment times in a district general hospital in London. Such an approach allowed decision-makers assessing its capability to meet the government target in regard to this metric. A simulation model is also used in Joshi et al. [137] for helping managers of a real emergency department to balance workload, reduce burnout and decrease patient waiting time. In this case, the patient flow was improved and the average wait dropped by 73.2%.

Equal number of contributions addressing the waiting time problem is based on single lean manufacturing (LM) applications (*n* = 17 papers; 32.07% of single-approached papers). For instance, Cookson et al. [159] pinpointed over 300 instances of waste along the ED patient journey by employing VSM. Such intervention helped healthcare leaders to improve the time to initial assessment. Generally speaking we also observe some papers that have validated the effectiveness of LM when facing the lengthy waiting times in EDs. Kane et al. [56] demonstrated that ED patient experience can be significantly improved by incorporating lean approaches. More recently, Sánchez et al. [63] applied lean thinking in triage acuity level-3 patients to improve waiting time of a tertiary hospital ED. As a result, significant reductions were achieved in waiting time (71 vs. 48 min, *p* < 0.001) and other critical measures.

The literature also reports a growing trend (*n* = 8 papers; 15.09%) in the use of OR methods (different from simulation) in a single form upon addressing lengthy door-to-treatment times in EDs. Oueida et al. [86] used petri nets for improving LOS, resource utilization, and patient waiting time in a real emergency care institution. Similar objectives were pursued by Bordoloi and Beach [151] who, unlike the previous work, used optimization models encompassing the entire patient journey within the ED. Single OR-based approaches are also extensively used in Ajmi et al. [83], Derni et al. [87], Leo et al. [153], Meng et al. [152], Nezamoddini and Khasawneh [154], and Oueida et al. [96]. Other non-hybrid methods that have been employed for tackling this ED deficiency are as follows: REACT [91], pivot nursing [92], process redesign [94,156], regression [157,158], nurse navigator [160], Iowa model of evidence-based practice [161], CQI [81,155], and ED dashboard/reporting [98].

### 3.3. Papers Focusing on Tackling the Overcrowding

Table 4 presents all the interventions focused on reducing overcrowding in EDs. As discussed in previous studies [7,8,9] and evidenced in this review, there is an increased interest on solving the overcrowding problem in EDs. Such interest is motivated by the negative effects that have been pinpointed in several congested EDs. These effects include delayed diagnosis and treatment, extended pain and suffering, and risk for poor outcomes. As the population ages and life expectancy augments, aggressive solutions are expected from practitioners and research community. In this regard, several studies have suggested a variety of process improvement approaches that can be also adopted by the emergency department directors for addressing this serious problem. In these studies, either a single approach (n = 32 papers; 58.18%) or a hybrid method (n = 23 papers; 41.81%) was proposed for counteracting this international issue.

Given the multifactorial origin and complexity of ED congestion, robust approaches are beginning to be often considered in the literature. Unsurprisingly, most of these approaches include simulation techniques (*n* = 13 papers; 54.16%). For example, some authors have proposed the integration of optimization models and simulation to determine the best bed allocations considering both tactical and operational decisions as exemplified in Landa et al. [199]. In this work, the simulation model represented the patient flows of a medium-size hospital ED located in Genova, Italy. The intervention was motivated by the increased congestion experience in this department and the growing concern on decreasing the number of inpatient ward beds. Similar applications using DES and optimization models can be found at Kuo [174] and Sinreich et al. [121]. Other studies expose the integration of simulation with BSC and PRIME [131], FAHP and MAUT [129], DGP [20], lean manufacturing [116,166], six sigma [198], DOE [111,124], approximation algorithm [103], and other OR methods [102] for reducing overcrowding within emergency departments. However, none of these integrations has been widely adopted in the ED context.

Different OR methods were also merged for addressing the overcrowding problem in EDs. Initially, Othman et al. [178] used multi-agent system along with multiskill task scheduling for helping physicians of a French pediatric ED to anticipate the feature of overcrowding. Another intervention using a mix of OR methods can be seen in El-Rifai et al. [196] where a two-stage stochastic integer linear program and sample average approximation were conjointly used for managing staff allocation and consequently coping with congestion in an ED located in Lille, France. Decreasing overcrowding by combining OR methods were also found in González et al. [172], Acuna, et al. [132], and He et al. [109]. Apart from these works, some authors proposed the use of lean six-sigma [194,195] and regression analysis [26,197,200].

Various methods were also employed separately by authors as an aid to reduce crowding in emergency departments. For example, the ability of simulation to model the multi-causality nature of ED overcrowding in a great level of detail makes this technique a potential tool for administrators and policy makers, even when employed in a single form. In fact, our review reports 12 papers (37.5%) evidencing the use of this technique in congested EDs. We noted that as Ahalt et al. [185] discuss, simulation can serve as a way of measuring crowdedness, a metric that avoids efforts being expanded on unnecessary interventions and guides administrators towards the design of cost-effective solutions. On the other hand, Fitzgerald [186] described how simulation has propelled cultural changes in congested Australian EDs through providing fast and accurate predictions on change outcomes. Since then, innovative studies endorsing the use of simulation in overcrowded EDs has been ample.

The use of lean manufacturing also continues to rise among researchers and practitioners who are concerned on systematically evaluating interventions as well as implementing evidence-base policies. In this review, 10 papers (31.25%) were found to offer solutions to the overcrowding problem after employing LM. A fruitful LM program is exposed in Van der Linden et al. [65] where after a 9-month intervention, the modified National ED Overcrowding Score (mNEDOCS) dropped from 18.6% to 3.5%. An earlier LM project is presented in Al Owad et al. [190] where voice of costumer, voice of process, and voice of staff were integrated for diminishing overcrowding in a hospital ED located in Saudi Arabia.

Regression applications are relatively new in the literature in relation to supporting improvements in busy emergency departments. Eiset et al. [158] adopted a transition regression model based on past departures and pre-specified risk factors to predict the expected number of departures and waiting time in the ED unit at Aarhus University Hospital (Denmark). The authors concluded that the number of arrivals has the biggest effect on departures with an odds ratio of 0.942. Multipronged efforts in tackling this problem were also demonstrated in Singh et al. [72] where a multivariate logistic regression model was developed considering four ED crowding scores, patient-related, system-related, and provider-related risk factors. Other contributing studies utilizing regression are available at Hu et al. [192] and Van der Veen et al. [74]. Less explored single approaches include: agent-based dynamic optimization [83], process redesign [94], Fulbrook et al. [160], integer programming [154], SCLP [193], and Iowa model of evidence-base practice [161].

### 3.4. Papers Focusing on Diminishing the Patient Flow Time in ED

The papers targeting decreased patient flow times within EDs are enlisted in Table 5. According to our review, lengthy patient flow time has received increasing attention due to its complexity and importance on clinical outcomes. Across many emergency care settings, patient flow problems have reached epidemic proportions. In fact, longer patient journey times are associated with patient dissatisfaction, more severe clinical complications, and increased mortality rates. The problem is even more sharpener considering the ineffective response of EDs to the growing demand of emergency care services. To substantially counteract this problem, several single (n = 45 articles; 63.38%) and integrated (n = 26 articles; 36.62%) approaches from different research fields have been proposed by authors.

As we will next briefly describe, the combined approaches have provided sustained support for restructuring patient flows within EDs. Most studies have emerged proposing the use of simulation as the cornerstone of several combined methodologies (*n* = 19 papers; 82.6%). In particular, the literature reports several studies mixing OR methods and simulation to cope with the patient flow problem. Zeinali et al. [184] used a simulation-based metamodeling approach to deal with patient’s congestion in an Iranian ED. The experimental outcomes confirmed that patient flow can be substantially improved with this approach even under budget and capacity constraints. The continuous strain caused by the increased number of emergency admissions also motivated Elalouf and Wachtel [103] to develop an approximation algorithm whose results were later embedded in a simulation procedure. Such procedure underpinned the design of cost-effective triage solutions facilitating the patient flow within an ED located in Israel. The problem here considered was extended by incorporating uncertainty inherent to the real-life scenario.

A few studies presented a comprehensive combination between simulation and lean to additionally eliminate non-value added activities along the ED patient journey. A tremendous effort, for instance, was documented in Huang and Klassen [216] who also incorporated six-sigma for improving the phlebotomy process in the ED of the St. Catharines Site of the Niagara Health System. Such integration led decision-makers to identify potential improvement opportunities and propose solutions with an estimated 7-minute flow time reduction. The amount of time spent in EDs was also evaluated in Romano et al. [116] through the combination of lean healthcare, simulation, and causal loop diagrams. This framework was implemented in an Italian ED where positive results in patients’ flow were further evidenced with subsequent reductions of profit loss. Scientific evidence also point out the presence of simulation-based hybrid approaches incorporating other less prominent techniques such as: fuzzy logic [212], what-if analysis [213], capability analysis [217], statistical methods [25], and decision-making [125]. In addition, a highlighted study is presented by Gartner and Padman [171] who integrated machine learning and DES to improve the patient flow of a real ED. The results revealed that changing staffing patterns can lead to shorter patient journey times.

Some investigators have tackled the patient flow problem through mixing other process-improvement methods. It is worth noting, for example, the use of lean manufacturing combined with quality management techniques. A related case is exposed by Stanton et al. [220] who implemented lean six-sigma for improving the patient flow from the ED to the wards of an Australian hospital. The LSS project also had significant positive impact on involved staff and resource leveraging. Similar lean-based hybrid applications can be also found at Ryan et al. [218] and Weimann [221]. To substantially redesign ED patient journey other authors preferred using integrated approaches including statistical methods [118,197] or only OR methods as cited in González et al. [172] and Lau et al. [175].

As evidenced above, a considerable percentage of the studies targeting reduced patient flow (63.6%) employed a single approach as a methodological basis. The most popular method used in a single way upon facing the patient flow challenge is lean manufacturing (13 papers; 28.88%). Dickson et al. [51] reported a 2-year experience of an academic emergency treatment center employing LM for continuously improving the patient flow. After implementation, the direct expense per patient has dropped by 9% (from US$112 to US$102.5) and patient satisfaction has increased by almost 10%. A similar work is seen in Matt et al. [203] where a LM program demonstrated to be beneficial for four different ED hospitals in Northern Italy. The results revealed that the patient lead-time from registration to discharge was significantly lessened by 17%.

Definitively, simulation is one of the most used techniques for underpinning improvements in emergency department even when employed separately. Door-to-discharge times are not the exception to this rule. A comprehensive simulation model implemented in Khanna et al. [201] confirms the previous statement. The DES model here designed was employed for evaluating operationally realistic scenarios on flow performance. As a result, the National Emergency Access Target (NEAT) performance increased by 16% whilst average bed occupancy diminished by 1.5%. Patient pathways from hospital presentation to discharge were also studied in Vile et al. [202] where a DES model was implemented for helping a major UK hospital ED to enhance the key ED performance target to admit or discharge 95% of patients within 4 h of arrival. This implementation has propelled the continuous use of simulation as a robust platform supporting the design of flexible EDs. Thereby, managers can establish whether the resources are well managed while providing high-quality emergency care to patients.

Another quality-related methodology found to offer solutions to the patient flow problem is CQI. Although most of this literature was published between 1996 and 2003, meaningful insights can be extracted by policy makers for addressing this burden properly. Goldmann et al. [204] presented a CQI program whose implementation led to a 71-minute reduction in the time from triage to discharge experienced by patients attending to a pediatric teaching hospital ED. Over the recent years, Preyde et al. [81] exposed a CQI program whose implementation led to a reduction of 1.16 h in the total time spent for patients admitted at a Canadian hospital ED. Other single techniques were used for tackling lengthy patient journey times within EDs; however, their application has been poorly explored as further evidenced throughout the literature. These include optimization models [83,151], petri nets [87,96], process redesign [94,156], mixed integer programming [208], nurse navigator [160], acute care model [209], critical pathways [210], fast track protocols [211], Iowa model of evidence-based practice [161], and regression analysis [75].

### 3.5. Papers Focusing on Diminishing the Number of Patients Who Leave the ED Without Being Seen

Table 6 depicts the articles focusing on diminishing the number of patients who leave the ED without being seen. Given the low number of papers contributing to this research field (*n* = 25 papers), we can conclude that improvement processes in this area are at the earlier stages and more interventions from research community are therefore expected for building a solid evidence base. Moreover, there is a great need for addressing the increased LWBS rates reported internationally [17] which, in the meantime, are associated with elevated readmission rates and patient dissatisfaction. Such deficiencies may result in reputational damage, profit loss, and other financial implications related to repeated episodes of presentation. Additionally, there is a potential risk of ambulance misuse considering that approximately a third of LWBS patients arrive by ambulance. In response, several initiatives based on single (*n* = 19 articles; 76.0%) and multi-methods (n = 6 articles; 24.0%) approaches. Unsurprisingly, simulation tools continue to be the most preferred technique in multi-methods approaches addressing the leading problems in emergency departments. For instance, simulation has been applied along with statistical methods to deal with the LWBS problem. This is the case exposed in Yousefi et al. [127] who integrated agent-based simulation and ordinary least squares regression for representing the behavior of patients leaving a public hospital emergency department. In this study, four preventive policies were pretested for minimizing the LWBS rate. After intervention, the average LWBS and ED-LOS diminished by 42.14% and 6.05% respectively. A similar research study is reported in Easter et al. [25] who used DES, ANOVA, linear regression, and non-linear regression for evaluating different improvement scenarios in terms of LWBS and other critical emergency care measures. The results evidenced that LWBS can decrease between 0.66% - 2% if an additional internal-waiting room is adopted within the emergency department. Much effort was also evidenced in papers integrating simulation with other approaches. For example, Lee et al. [112] coupled machine learning, simulation, and optimization to reduce the number of patients who leave without being seen in the ED at Grady Memorial Hospital (Atlanta, Georgia). As a result, the LWBS was reduced by more than 30% along with cost savings and annual revenue of approximately $190 million. The rest of studies based on integrated methods used a combination of statistical methods [222] and a mix of OR techniques [125,223] for tackling elevated LWBS and their consequences mainly affecting the financial sustainability of EDs.

In general, single methods were found to be most popular compared to hybrid approaches when targeting minimized LWBS. International evidence reveals that most of research studies focused on this problem used a quality-improvement approach (*n* = 16 papers; 84.21%). These approaches have provided an excellent step forward in counteracting the LWBS causes by removing special causes of variation, non-value added activities, and unpleasant environment conditions in waiting rooms. Evidently, the most prominent technique was Lean Manufacturing (*n* = 11 papers; 57.89%) which entails a variety of tools perfectly addressing the above-mentioned causes. The first related contribution was presented by Dickson et al. [52] who described the lean effects on the percentage of patients who left without being seen associated with two hospital EDs. After 1 year post-lean the LWBS in the hospital A dropped from 8% to 5% while hospital B experienced a 22% decrease after 3 years of implementation. More recently, Peng et al. [60] used lean healthcare for reducing the LWBS rates of rural EDs. After intervention, this metric was reduced from 4.1% to 2.0% (*p* < 0.001) while LOS was also significantly diminished with a *p* < 0.001.

Another quality-improvement approach found to address the left-without-being-seen rates was CQI. In particular, Rothwell et al. [155] struggled to manage this problem in an Arabic ED by implementing a 3-month quality improvement project including a new fast-track unit. A longer project is observed in Preyde et al. [81] where a 6-month process improvement program was applied for reducing LWBS patients of a Canadian hospital ED. After implementation, fewer patients (*n* = 425) left without being seen was reported along with additional improvements in other important emergency care metrics. Other studies using CQI-based implementations for addressing this problem can be found at Rehmani and Amatullah [82] and Welch and Allen [224]. Aside from the above-cited single methods, investigators have employed REACT, pivot nursing, process redesign, and statistical process control as correspondingly evidenced in Chan et al. [91], Christensen et al. [92], DeFlitch et al. [94], and Schwab et al. [225]. Surprisingly, simulation tools have not used in a single way for coping with this problem and its side effects.

## 4. Discussion

Our review reveals a considerable growth in the number of papers exposing process improvement methodologies addressing the main problems reported in EDs. In particular, the increasing publication trend initiated around 2011 concentrates 84.23% of the total related scientific contribution (*n* = 171 papers). This, of course, evidences the growing interest of policy makers, ED administrators, decision makers, researchers, and practitioners in this research field and the latent need for providing a high-quality and sustainable emergency care to patients. This is also consistent with the recent bunch of interventions that have been propelled by governments from different countries (as the 4-hour target – NEAT – established by the UK) searching for reducing mortality and morbidity rates, cost overruns, and adverse events. On the other hand, most of the evidence base is provided by journals from medical sciences, operations research, and quality fields, which demonstrates the multidimensional nature of ED context and the wide variety of process improvement approaches that can be used by ED administrators when facing the ED problems cited in this review.

**Figure 4:**
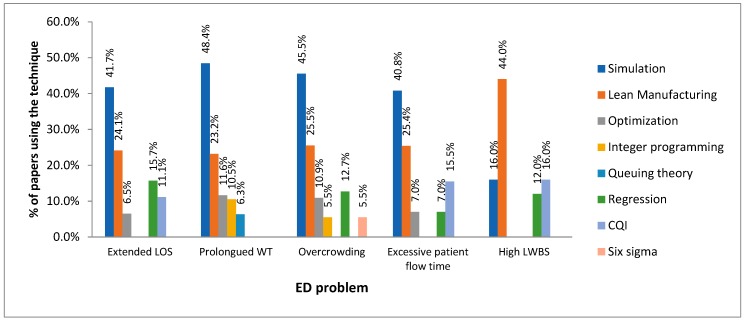
The most prominent techniques used for addressing the top-five leading problems in EDs.

One of the major findings from the review is the prominent use of simulation and LM techniques in the solution of ED deficiencies (Figure 4). The only exception was evidenced in *High LWBS* where LM was found as the most preferred approach. Authors have mostly employed this approach since: i) it provides a reliable representation of the patient journey within EDs so that factors and interactions affecting emergency care can be easily identified, ii) it records individual entity experience which is desirable for analyzing inefficiency patterns, iii) it facilitates engagement with decision-makers through animation, and iv) it allows ED managers to pretest potential improvement scenarios [226,227,228,229]. It is also noteworthy that researchers have decided to utilize lean manufacturing preferentially since it i) allows ED managers identifying and removing the causes of emergency care variability, thus minimizing prolonged stays within these departments, ii) enables managers to detect and reduce wastes of resources (including time and cost overruns), iii) increases patient satisfaction rates, and iv) promotes collaborative work and increases the competences of medical staff. Another major benefit of LM is the ability to reduce the service lead time by adopting standard operating procedures that diminish expenses, increase efficiency, and improve operations. Lean thinking, as a bunch of concepts and tools directed towards the operational excellence, empowers medical and administrative staff to continuously identify significant opportunities in the ED which ends up increasing their technical competences whilst leading to a sustainable reduction of patient flow time, behavioral changes, and increased throughput. On a different note, the simplicity and efficiency of Queuing theory endorses its application on improving the emergency care experienced by ED patients. Also, the use of optimization techniques is a desired alternative when decision-makers need to maximize the impact of investments (for example, minimizing ED-LOS) under constrained resources as often observed in public EDs.

We also noted that 36 different methods have been employed by authors for dealing with the excessive stays in emergency department. To date, most of work has focused on the use of OR methods. This is validated by the presence of simulation (*n* = 45 papers = 41.7%), optimization (*n* = 7 papers = 6.5%), and queuing theory (*n* = 5 papers = 4.6%) in the top-five of most popular techniques. On a different tack, quality improvement techniques can be also highlighted as a good option for addressing this problem. For instance, some authors are skewed to continuous quality improvement interventions (*n* = 10 papers = 9.3%) given their easy adoption by administrative and clinical staff, patient centered nature, and ability of constantly upgrading ED performance (as expected with LOS and other critical ED measures). Surprisingly, regression (*n* = 17 papers = 15.7%) was ranked third in the list of popular improvement tools. This technique has been often applied due to its ability of evidencing improvement or decline in key operational variables (such as LOS). It is clear from these findings that there is much room for the application of combined approaches considering the most popular OR (simulation, queuing theory, and optimization), regression, and quality improvement (lean manufacturing, CQI) techniques which is highly suggested for ED managers, decision-makers, practitioners, and researchers when dealing with long stays in emergency care settings. Such integration lays the groundwork for implementing a high-performance system-wide approach that would greatly lower ED stays even in the presence of growing and peak demands. In addition to this research opportunity, the reported literature revealed various gaps that should be properly addressed within the upcoming interventions targeting shortened LOS: (i) There are only a few initiatives considering data-driven approaches and behavioral aspects of emergency care, (ii) There is no reported literature concerning how LOS can be reduced in emergency care networks, (iii) There are no case studies considering patient heterogeneity and multiple care options, (iv) Only few works contemplate the participation of EDs, government, and academic sector in the design of improvement strategies shortening ED LOS.

On a different tack, 48 different techniques have been utilized by authors for coping with the lengthy door-to-doctor times in emergency departments. Most of the research has been skewed to the application of OR methods as observed in interventions reducing LOS. In fact, four OR methods were listed among the six most popular approaches: simulation (*n* = 46 articles = 48.4%), optimization (*n* = 11 articles = 11.6%), integer programming (*n* = 10 articles = 10.5%), and queuing theory (*n* = 6 articles = 6.3%). An interesting finding is related to the use of integer programming for decreasing the door-to-treatment times. The increasing use of this method is founded on its ability to achieve near optimal solutions in a realistic time frame. On the other hand, it is seen that some practitioners have preferred using lean manufacturing (*n* = 22 articles = 23.2%) and regression (*n* = 5 articles = 5.3%) for reducing waiting times within EDs as similarly found in the previous ED problem. Moreover, 43 interventions targeting shortened ED stays were simultaneously directed towards the improvement of door-to-treatment times. The above-mentioned findings endorse the integration of these methods as a powerful and robust framework addressing extended waiting times and lengthy stays in emergency departments. This approach is then highly attractive and useful for decision-makers considering their need for allocating scarce resources in high-impact solutions. There are, however, very few studies evidencing the use of hybrid methods for this particular aim. The reported related literature also revealed that data-driven approaches were not considered when tackling the waiting time problem. Besides, there is no research dealing with this phenomenon in emergency care networks. Therefore, future efforts in this research field should be directed towards the aforementioned lines.

It is also noteworthy that 30 different methods have been used by researchers and practitioners to deal with ED “admission hold”. A great portion of the interventions has mostly adopted OR methods as also observed in the above-cited ED problems. In this case, three OR methods were ranked among the most prominent approaches: simulation (*n* = 25 articles = 45.5%), optimization (*n* = 6 articles = 10.9%), and integer programming (*n* = 3 articles = 5.5%). We also see a high percentage of research considering lean thinking (*n* = 14 articles = 25.5%) and regression models (*n* = 7 articles = 12.7%) for tackling ED overcrowding as also detected in the previous ED problems. The multifaceted nature of these approaches is then attractive for ED directors, administrators, and policy makers who search for methodological frameworks able to address different problems at once. This is motivated by the need for continuously providing urgent care and allocating scarce resources properly. It is also important to stress the inclusion of six-sigma as an alternative for minimizing process variability in supporting services like radiology and laboratory which often contribute to ED congestion. In light of these facts, combining all these techniques can be a fruitful path for research and interventions underpinning the day-to-day management of ED congestion. On a broader scale, decisions such as hiring or firing new doctors or nurses, buying new beds and building new observation rooms can be properly assessed through the use of these methodologies. Other gaps detected in the related literature are as follows: (i) A small number of interventions are related to overcrowding in developing countries, (ii) The methodological approaches here cited do not consider patient heterogeneity and multiple care options, and (iii) Most overcrowding-related case studies do not evidence close collaborations amongst academic sector, government, and EDs.

Not coincidentally, the presence of OR (simulation and optimization), quality-improvement (lean manufacturing and CQI) and regression techniques was also evidenced in studies targeting reduced door-to-discharge times in EDs. Using the aforedescribed methods in a combined approach may be then useful for administering patient flows robustly. These methods can suitably deal with an operational context compounded by multiple transient stages, interactions, treatment alternatives, and outcomes. Thereby, decision makers may better predict the potential impact of demand changes and ED configurations on downstream operations, critical emergency care measures, and financial metrics of interest. Other research challenges related to this problem are the following: (i) The implementation of data-driven approaches (i.e., data mining, process mining) combined the large amount of data derived from emergency care, (ii) The replication of the aforementioned interventions in developing countries where the financial budget is highly restricted, and (iii) The application of multi-phase models that better represent the multifactorial context of emergency care while outlining the interrelations with other healthcare services (i.e., hospitalization, surgery, intensive care unit, radiology).

The review also led to identify the variety of process-improvement methods (*n* = 15) that have been trialed for reducing the left-without-being-seen rates in different countries. In this case, lean manufacturing (*n* = 11 papers; 44.0% out of the total contributions) was found to be the most prominent technique when addressing this problem. The second place in the rank is shared by CQI (*n* = 4 papers = 16% of the total contributions) and computer simulation (*n* = 4 papers = 16% of the total contributions) while regression (*n* = 3 paper = 12.0%) was also listed among the most popular approaches addressing elevated LWBS rates. This evidence supports the integration of simulation approaches and process improvement techniques originated from the automotive industry (such as LM and CQI) in an effort to improving several critical emergency care measures (i.e., average LWBS) [10]. A concern, however, is the availability of high-quality and suitable data, an aspect also pointed out in Clarey and Cooke [17]. Modelers require detailed and intricate data for providing a good representation of patient pathways directly affecting ER waiting times, one of the major factors associated with high LWBS rates. Decision makers should then establish strategies for ensuring proper data collection underpinning the deployment of the aforementioned combined approach. As discussed above, this research field is at the earlier stages and more advanced contributions are hence expected for expanding the evidence base of improvements addressing this problem. Apart from the previous considerations, future investigations should consider the inclusion of behavioral aspects explaining the LWBS rates. Moreover, more interventions are needed in developing countries where this problem has reached desperate proportions [226].

Our vision is also consistent with the WHO document entitled as “Delivering quality health services: A global imperative for universal health coverage” [230] which reinforces the need for the continuous collaboration between EDs, government, and academic partners for ensuring scale-up and sustainable improvement interventions in emergency care. The techniques here described will serve as a platform for interventions focused on upgrading the emergency care performance in terms of lead-time, equity, coordination, and efficiency as pursued by WHO. It is, however, critical to tackle some general methodological limitations that became evident from the literature. For instance, the use of hybrid approaches emerging from the combination of several prominent approaches is at the earlier stages and more contributions are then expected to increase the evidence base related to these applications. In particular, the use of combined interventions using simulation and lean manufacturing remains limited in the reported literature [113,115,116,166,176,216]. Likewise, researchers are advised to take into account the methodological trends regarding process improvement in emergency departments. For example, over the recent years, there has been a growing tendency to undertake multi-objective interventions as cited in Easter et al. [25] and Ajmi et al. [83]. Furthermore, there has been a downward trend in recent years concerning the use of CQI-based approaches which may be explained by the adoption of more robust approaches like LM. By considering the findings discussed in this section, decision-makers and other stakeholders can better define short-term and long-term improvement plans pursuing high-quality emergency care and reduced operational cost whereas providing new evidence base for the development of more effective interventions and research.

## 5. Concluding Remarks and Future Directions

A wide variety of process improvement methodologies have been employed by researchers and practitioners for addressing leading emergency department inefficiencies including Overcrowding, Prolonged waiting time, extended length of stay (LOS), excessive patient flow time, and High number of patients who leave the ED without being seen (LWBS). In order to lay groundwork for devising and implementing cost-effective solutions as well as detecting limitations in current practice, this paper provided a comprehensive literature review comprising of 203 papers spread over the period ranged between April 1993 and October 2019. The papers, distributed in 120 journals, were then examined and classified according to the: (i) targeted ED problem and (ii) publication year. We also identified the most prominent process-improvement approaches that have been used for tackling each of the aforementioned ED deficiencies. In particular, we particularly noted that process-improvement studies in EDs are ample when coping with prolonged waiting time, extended LOS, and excessive patient flow time; nonetheless, there is still a lack of interventions tackling overcrowding and high left-without-being-seen rates. This is mainly caused by the poor involvement of ED administrators, policy makers, and other stakeholders in the design of multifaceted suitable strategies addressing the complexity and implementation conditions inherent to the real ED context.

It is noteworthy that simulation has been the most popular approach for addressing the leading operational problems due to their capability to deeply analyse the current performance of emergency services, pre-test improvement scenarios, and facilitate user engagement through the animation of patient flows and resources. Lean manufacturing, regression analysis, optimization, and CQI were also found to be highly used by practitioners and researchers when addressing the ED deficiencies. In particular, authors employed OR methods (simulation and optimization), quality-improvement techniques (lean manufacturing and CQI), and regression for tackling extended patient flow times and lengthy ED stays. On a different tack, researchers utilised lean manufacturing, simulation, optimization, regression, and integer programming for addressing overcrowded emergency departments. Meanwhile, CQI, lean manufacturing, simulation, and regression were mostly used for decreasing the left-without-being-seen rates. However, we look for hybrid approaches using these methods for fully exploiting the advantages of each technique so that more robust results can be achieved in the real-life scenario.

Unsurprisingly, the application of single approaches is more widespread compared to integrated techniques when addressing the above-mentioned ED problems. There is, however, a growing trend in the use of hybrid methods justified by the complexity of emergency care operations, the interactions with other services, and the continued increased demand. There are no, however, studies combining simulation, lean manufacturing, optimization, CQI, and regression for tackling any of the leading ED problems. Both combinations are projected to effectively underpin ED operations for delivering optimized emergency care under reasonable costs. Therefore, such approaches are expected to be fruitful paths for future research.

There are also a limited number of studies addressing different emergency department deficiencies at once. Hence, more similar contributions are expected to expand the current research body and widespread the use of these approaches in real-life EDs. Furthermore, there is a definite need for implementing these methods in emergency care networks (ECNs) to identify key lessons underpinning the deployment of effective and timely ECNs in the future. We also expect to see more advancement regarding the use of data-driven approaches considering behavioral aspects inherent to emergency care. Thereby, more realistic and representative models can be designed for supporting multifaceted interventions encompassing upstream services.

In conclusion, future research should be directed towards: (i) more contributions integrating simulation and lean manufacturing, (ii) studies combining optimization, CQI, lean manufacturing, simulation, and regression, (iii) interventions based on data-driven approaches and behavioral aspects of emergency services, (iv) implementations of process improvement methodologies underpinning emergency care networks, (v) more projects addressing different emergency department problems at once, vi) interventions tackling overcrowding and high left-without-being-seen rates, (vii) the design and implementation of new modelling frameworks considering patient heterogeneity and the multiple care options with the goal of underpinning the deployment of strategic plans within emergency care and its associated services, viii) the promotion of international collaboration to develop comparative studies among countries and new guidelines for process improvement, (ix) propel the widespread application of the identified approaches in developing countries where financial budget is largely limited, (x) foster closest collaborations among EDs, government, and academic partners for designing scale-up and sustainable improvement interventions in emergency care, (xi) review research progress related to interventions addressing non-urgent ED admissions considering the high waste of resources reported by hospitals and clinics, especially on weekends, and (xii) review the literature regarding improvement strategies including clinical-related interventions, personnel training, the ABCDE of Emergency care, and Triage which have not been covered in this paper. If properly addressed, these research lines will provide decision makers with a potent decision-making platform for effectively facing the expected growing demand at a minimum operational cost.

## Figures and Tables

**Table 1:** Classification of papers according to the targeted ED problem and publication year.

Period	*N* (Papers/Period)	Extended LOS	Prolonged Waiting Time	Excessive Patient Flow Time in ED	Overcrowding	High LWBS
1993–2004	11 (5.41%)	4	2	8	0	1
2005–2006	5 (2.46%)	2	2	0	1	2
2007–2008	7 (3.44%)	3	3	3	0	1
2009–2010	9 (4.43%)	8	2	2	1	2
2011–2012	26 (12.80%)	14	19	8	7	3
2013–2014	20 (9.85%)	10	6	10	9	1
2015–2016	34 (16.74%)	17	21	12	10	5
2017–2018	64 (31.52%)	34	22	19	18	5
2019	27 (13.30%)	16	18	9	9	5
*N* (papers/problem-period)		108	95	71	55	25
Participation (%)		53.20	46.79	34.97	27.09	12.31

**Table 2:** Papers evidencing the use of process improvement techniques for shortening LOS within EDs.

Authors	Technique Type
Single	
Ajdari et al. [27]; Best et al. [28]; Bokhorst and van der Vaart [29]; Coughlan, Eatock, and Patel [30]; Gul and Guneri [31]; Hung and Kissoon [32]; Ibrahim et al. [33]; Keyloun, Lofgren, and Hebert [34]; Khare et al. [35]; Konrad et al. [36]; La and Jewkes [37]; Medeiros et al. [38]; Oh et al. [39]; Paul and Lin [40]; Rasheed et al. [41]; Rosmulder et al. [42]; Saoud, Boubetra, and Attia [43]; Steward, Glass, and Ferrand [44]; Thomas Schneider et al. [45]; Wang et al. [46]; Zeng et al. [47]	Simulation or Discrete-event simulation (DES)
Allaudeen et al. [48]; Arbune et al. [49]; Carter et al. [50]; Dickson et al. [51,52,53]; Elamir [54]; Hitti et al. [55]; Kane et al. [56]; Migita et al. [57]; Murrell, Offerman, and Kauffman [58]; Ng et al. [59]; Peng, Rasid, and Salim [60]; Polesello et al. [61]; Rotteau et al. [62]; Sánchez et al. [63]; Sayed et al. [64]; Van der linden et al. [65]; Vermeulen et al. [66]; White et al. [67]	Lean manufacturing
Cheng et al. [68]; Forero et al. [69]; Kaushik et al. [70]; Maniaci et al. [71]; Singh et al. [72]; Street et al. [73]; Van der Veen et al. [74]; Yau et al. [75];	Regression
Brent et al. [76]; Fernandes and Christenson [77]; Fernandes, Christenson, and Price [78]; Higgins III and Becker [79]; Lovett et al. [80]; Preyde, Crawford, and Mullins [81]; Rehmani and Amatullah [82]	Continuous quality improvement
Ajmi et al. [83];	Agent-based dynamic optimization
Haydar, Strout, and Baumann [84]; Prybutok [85]	PDSA (Plan, Do, Study, Act) cycle
Oueida et al. [86]; Derni, Boufera, and Khelfi [87]	Petri nets
Bellew et al. [88]; Than et al. [89]	Critical pathways
Brouns et al. [90]	Cohort study
Chan et al. [91]	Rapid Entry and Accelerated Care at Triage (REACT)
Christensen et al. [92]	Pivot nursing
Christianson et al. [93]	Six sigma
DeFlitch et al. [94]	Process redesign
Liu et al. [95]	Agent-based model
Oueida et al. [96]	Resource Preservation Net (RPN)
Sloan et al. [97]	Evidence-base care pathways
Stone-Griffith et al. [98]	ED dashboard and reporting application
Hybrid	
Ashour and Kremer [20]	Dynamic grouping and prioritization (DGP), Discrete-event simulation
Bish, McCormick, and Otegbeye [99]	Simulation, Queuing analyses
Blick [100]	Lean Six Sigma
Chadha, Singh, and Kalra [101]	Lean manufacturing, Queuing theory
Chen and Wang [102]	Non-dominated sorting particle swarm optimization (NSPSO), Multi-objective computing budget allocation (MOCBA), Discrete-event simulation
Easter et al. [25]	Discrete-event simulation, Analysis of Variance (ANOVA), Linear regression, Non-linear regression
Elalouf and Wachtel [103]	Approximation algorithm, Simulation
Feng, Wu, and Chen [104]	Non-dominated sorting genetic algorithm II (NSGA II), Multiple computing budget allocation (MOCBA), Discrete-event simulation
Ferrand et al. [105]	Simulation, Dynamic priority queue (DPQ)
Fuentes et al. [26]	Logistic regression, Linear regression, Paired t test, Wilcoxon signed rank
Furterer [106]	Lean Six Sigma
Ghanes et al. [107]	Optimization, Discrete-event simulation
Goienetxea Uriarte et al. [108]	Discrete-event simulation, Simulation-based multi-objective optimization, Data mining
He, Sim, and Zhang [109]	Mixed integer programming, Queuing network, Stochastic Programming
Huang et al. [110]	Descriptive statistics, Two-sample t-test, Multivariate linear regression
Kaner et al. [111]	Discrete-event simulation, Design of experiments
Lee et al. [112]	Machine learning, Simulation, Optimization
Lo et al. [113]	Lean principles, Simulation, Continuous process improvement
Oueida et al. [114]	Discrete-event simulation, Optimization
Rachuba et al. [115]	Process mapping, Discrete-event simulation
Romano, Guizzi, and Chiocca [116]	System dynamics simulation, Lean techniques, Causal loop diagram
Ross, Johnson, and Kobernick [117]	Critical pathways, Continuous quality improvement
Ross et al. [118]	Multivariate logistic regression, Ordinary least squares regression
Shin et al. [119]	Discrete-event simulation, Linear integer programming
Sinreich and Jabali [120]	Linear optimization model (S-model), Heuristic iterative simulation based algorithm
Sinreich, Jabali, and Dellaert [121]	Discrete-event simulation, Optimization
Sir et al. [122]	Classification and regression trees, Mixed integer programming
Techar et al. [123]	Multivariate logistic regression, Negative binomial models
Visintin, Caprara, and Puggelli [124]	Simulation, Experimental design
Yousefi and Ferreira [125]	Agent-based simulation, Group Decision Making
Yousefi et al. [126]	Agent-based simulation, Chaotic genetic algorithm, Adaptive boosting (AdaBoost)
Yousefi et al. [127]	Agent based modeling, Ordinary least squares regression
Zeltyn et al. [128]	Simulation, Queuing theory

**Table 3:** Articles evidencing the use of process improvement techniques for minimizing the ED waiting time.

Authors	Technique Type
Single	
Coughlan, Eatock, and Patel [30]; Duguay and Chetouane [135]; Hung and Kissoon [32]; Ibrahim et al. [33,136]; Joshi, Lim, and Teng [137]; Kaushal et al. [138]; Konrad et al. [36]; Lamprecht, Kolisch, and Pförringer [139]; Medeiros et al. [38]; Paul and Lin [40]; Rasheed et al. [41]; Saoud, Boubetra, and Attia [43]; Taboada et al. [140]; Wang et al. [141]; Yang et al. [142]; Zeng et al. [47]	Simulation or Discrete-event simulation
Carter et al. [50]; Elamir [54]; Hogan, Rasche, and Von Reinersdorff [143]; Ieraci et al. [144]; Improta et al. [145]; Kane et al. [56]; Murrell, Offerman, and Kauffman [58]; Ng et al. [59]; Piggott et al. [146]; Rees [147]; Rutman et al. [148]; Sánchez et al. [63]; Sayed et al. [64]; Vashi et al. [149]; Vermeulen et al. [66]; White et al. [150];	Lean manufacturing
Ajmi et al. [83]; Bordoloi and Beach [151]; Meng et al. [152];	Optimization
Leo et al. [153]; Nezamoddini and Khasawneh [154]	Integer programming
Queuing theory	
Preyde, Crawford, and Mullins [81]; Rothwell, McIltrot, and Khouri-Stevens [155]	Continuous quality improvement
DeFlitch et al. [94]; Spaite et al. [156]	Process redesign
Derni, Boufera, and Khelfi [87]; Oueida et al. [86]	Petri nets
Doupe et al. [157]; Eiset, Kirkegaard, and Erlandsen [158]	Regression
Chan et al. [91]	Rapid Entry and Accelerated Care at Triage (REACT)
Christensen et al. [92]	Pivot nursing
Cookson et al. [159]	Value Stream Mapping (VSM)
Fulbrook, Jessup, and Kinnear [160]	Nurse navigator
Oueida et al. [96]	Resource Preservation Net (RPN)
Popovich et al. [161]	Iowa Model of Evidence-Based Practice
Stone-Griffith et al. [98]	ED dashboard and reporting application
Hybrid	
Abo-Hamad and Arisha [131]	Simulation, Balance Scorecard (BSC), Preference ratios in multi-attribute evaluation (PRIME)
Acuna, Zayas-Castro, and Charkhgard [132]	Mixed integer programming, game theory, single and bi-objective optimization models
Ala and Chen [133]	Integer programming, Tabu search, L-shaped algorithm, Discrete-event simulation
Aminuddin, Ismail, and Harunarashid [162]	Simulation, Data Envelopment Analysis (DEA)
Andersen et al. [163]	Integer linear programming, Markov models, Discrete-event simulation
Aroua and Abdulnour [130]; Zhao et al. [164]	Simulation, Design of experiments (DOE)
Ashour and Kremer [20]	Dynamic grouping and prioritization (DGP), Discrete-event simulation
Azadeh et al. [165]	Mixed integer linear programming, Genetic algorithm (GA)
Bal, Ceylan, and Taçoğlu [166]	Value Stream Mapping (VSM), Discrete-event simulation
Benson and Harp [167]	Discrete-event simulation, System thinking
Bish, McCormick, and Otegbeye [99]	Simulation, Queuing analyses
Daldoul et al. [168]	Stochastic mixed integer programming, Sample average approximation
Diefenbach and Kozan [169]	Simulation, Optimization
Easter et al. [25]	Discrete-event simulation, ANOVA, Linear regression, Non-linear regression
EL-Rifai et al. [170]	Stochastic mixed-integer programming, Sample average approximation, Discrete-event simulation
Ferrand et al. [105]	Simulation, Dynamic priority queue (DPQ)
Gartner and Padman [171]	Discrete-event simulation, Machine learning
Ghanes et al. [107]	Optimization, Discrete-event simulation
Goienetxea Uriarte et al. [108]	Discrete-event simulation, Simulation-based multi-objective optimization, Data mining
González et al. [172]	Markov decision process, Approximate dynamic programming
He, Sim, and Zhang [109]	Mixed integer programming, Queuing network, Stochastic Programming
Izady and Worthington [173]	Discrete-event simulation, Queuing models, Heuristic Staffing Algorithm
Kuo [174]	Simulation-optimization
Lau et al. [175]	Genetic algorithm, Cost-optimization model
Martínez et al. [176]	Discrete-event simulation, Lean manufacturing
Mazzocato et al. [177]	Lean manufacturing, ANOVA
Othman et al. [178]	Multi-agent system, Multiskill task scheduling
Othman and Hammadi [179]	Fuzzy logic, Evolutionary algorithm
Oueida et al. [114]; Sinreich, Jabali, and Dellaert [121]	Discrete-event simulation, Optimization
Perry [180]	Lean manufacturing, Code critical
Romano, Guizzi, and Chiocca [116]	System dynamics simulation, Lean techniques, Causal loop diagram
Sir et al. [122]	Classification and regression trees, Mixed integer programming
Stephens and Broome [181]	Univariate analysis, Multivariate general linear regression, Binary logistic regression
Umble and Umble [182]	Theory of constraints, Buffer management, Synchronous management
Visintin, Caprara, and Puggelli [124]	Simulation, Experimental design
Xu and Chan [183]	Simulation, Queuing, Predictive models
Yousefi and Ferreira [125]	Agent-based simulation, Group Decision Making
Yousefi and Yousefi [134]	Agent-based simulation, Adaptive neuro-fuzzy inference system (ANFIS), Feed forward neural network (FNN), Recurrent neural network (RNN)
Zeinali, Mahootchi, and Sepehri [184]	Discrete-event simulation, Metamodels, Cross validation
Zeltyn et al. [128]	Simulation, Queuing theory

**Table 4:** Articles evidencing the use of process improvement techniques for tackling the ED overcrowding.

Authors	Technique Type
Single	
Ahalt et al. [185]; Ajmi et al. [83]; Best et al. [28]; Fitzgerald et al. [186]; Hung and Kissoon [32]; Ibrahim et al. [33,136]; Paul and Lin [40]; Peck et al. [187]; Rasheed et al. [41]; Restrepo-Zea et al. [188]; Thomas Schneider et al. [45]; Yang et al. [142]	Simulation or Discrete-event simulation
Aaronson, Mort, and Soghoian [189]; Al Owad et al. [190]; Elamir [54]; Hitti et al. [55]; Migita et al. [57]; Murrell, Offerman, and Kauffman [58]; Van der linden et al. [65]; Vose et al. [191]; White et al. [67,150]	Lean manufacturing
Nezamoddini and Khasawneh [154]	Integer programming
Eiset, Kirkegaard, and Erlandsen [158]; Hu et al. [192]; Singh et al. [72]; Van der Veen et al. [74]	Regression
Popovich et al. [161]	Iowa Model of Evidence-Based Practice
Wang [193]	Separated continuous linear programming (SCLP)
Fulbrook, Jessup, and Kinnear [160]	Nurse navigator
DeFlitch et al. [94]	Process redesign
Hybrid	
Abo-Hamad and Arisha [131]	Simulation, Balance Scorecard (BSC), Preference ratios in multi-attribute evaluation (PRIME)
Acuna, Zayas-Castro, and Charkhgard [132]	Mixed integer programming, game theory, single and bi-objective optimization models
Aldarrab et al. [194]	Lean Six Sigma
Ashour and Kremer [129]	Fuzzy Analytic Hierarchy Process (FAHP), Multi-attribute Utility Theory (MAUT), Discrete-event simulation
Ashour and Kremer [20]	Dynamic grouping and prioritization (DGP), Discrete-event simulation
Bal, Ceylan, and Taçoğlu [166]	Value Stream Mapping (VSM), Discrete-event simulation
Beck et al. [195]	Lean Six Sigma
Chen and Wang [102]	Non-dominated sorting particle swarm optimization (NSPSO), Multi-objective computing budget allocation (MOCBA), Discrete-event simulation
Elalouf and Wachtel [103]	Approximation algorithm, Simulation
El-Rifai, Garaix, and Xie [196]	Integer linear program (ILP), Sample Average Approximation (SAA)
Fuentes et al. [26]	Logistic regression, Linear regression, Paired t test, Wilcoxon signed rank
Garrett et al. [197]	Regression analysis, Vertical split flow
González et al. [172]	Markov decision process, Approximate dynamic programming
He, Sim, and Zhang [109]	Mixed integer programming, Queuing network, Stochastic Programming
Hussein et al. [198]	Six Sigma, Discrete-event simulation
Kaner et al. [111]	Discrete-event simulation, Design of experiments
Kuo [174]	Simulation-optimization
Landa et al. [199]	Multi-objective optimization, Discrete-event simulation
Othman et al. [178]	Multi-agent system, Multiskill task scheduling
Peltan et al. [200]	Multivariate regression, Markov multistate models
Romano, Guizzi, and Chiocca [116]	System dynamics simulation, Lean techniques, Causal loop diagram
Sinreich, Jabali, and Dellaert [121]	Discrete-event simulation, Optimization
Visintin, Caprara, and Puggelli [124]	Simulation, Experimental design

**Table 5:** Articles evidencing the use of process improvement techniques for minimizing patient flow time within EDs.

Authors	Technique Type
Single	
Coughlan, Eatock, and Patel [30]; Joshi, Lim, and Teng [137]; Khanna et al. [201]; Konrad et al. [36]; Lamprecht, Kolisch, and Pförringer [139]; Rasheed et al. [41]; Thomas Schneider et al. [45]; Vile et al. [202]; Yang et al. [142]; Zeng et al. [47]	Simulation or Discrete-event simulation
Al Owad et al. [190]; Dickson et al. [51]; Elamir [54]; Ieraci et al. [144]; Improta et al. [145]; Matt, Arcidiacono, and Rauch [203]; Ng et al. [59]; Rees [147]; Rotteau et al. [62]; Sánchez et al. [63]; Vermeulen et al. [66]; Vose et al. [191]; White et al. [67];	Lean Manufacturing
Fernandes and Christenson [77]; Fernandes, Christenson, and Price [78]; Goldmann et al. [204]; Henderson et al. [205]; Jackson and Andrew [206]; Lovett et al. [80]; Markel and Marion [207]; Preyde, Crawford, and Mullins [81];	Continuous quality improvement
Ajmi et al. [83]; Bordoloi and Beach [151]	Optimization
Yau et al. [75]	Regression models
Courtad et al. [208]	Mixed integer programming,
DeFlitch et al. [94]; Spaite et al. [156]	Process redesign
Derni, Boufera, and Khelfi, M [87]	Colored petri net
Fulbrook, Jessup, and Kinnear [160]	Nurse navigator
Haydar, Strout, and Baumann [84]	PDSA (Plan-do-study-act) cycle
Iyer et al. [209]	Acute care model
Mohan et al. [210]	Critical pathways
Ollivere et al. [211]	Fast track protocols
Oueida et al. [96]	Resource Preservation Net (RPN)
Popovich et al. [161]	Iowa Model of Evidence-Based Practice
Hybrid	
Ala and Chen [133]	Integer programming, Tabu search, L-shaped algorithm, Discrete-event simulation
Andersen et al. [163]	Linear programming, Discrete-event simulation
Azadeh et al. [212]	Fuzzy logic, Simulation
Benson and Harp [167]	Discrete-event simulation, System thinking
Bish, McCormick, and Otegbeye [99]	Simulation, Queuing analyses
Brenner et al. [213]	Simulation, What-if analysis
Diefenbach and Kozan [169]	Simulation, Optimization
Easter et al. [25]	Discrete-event simulation, ANOVA, Linear regression, Non-linear regression
Elalouf and Wachtel [103]	Approximation algorithm, Simulation
Ferrand et al. [105]	Simulation, Dynamic priority queue (DPQ)
Garrett et al. [197]	Regression analysis, Vertical split flow
Gartner and Padman [171]	Discrete-event simulation, Machine learning
González et al. [172]	Markov decision process, Approximate dynamic programming
Guo et al. [214]	Random boundary generation with feasibility detection (RBG-FD), Discrete-event simulation
Hajjarsaraei, Shirazi, and Rezaeian [215]	Discrete-event simulation, System dynamics
Huang and Klassen [216]	Six Sigma, Lean manufacturing, Simulation
Keeling, Brown, and Kros [217]	Capability analysis, simulation
Lau et al. [175]	Genetic algorithm, Cost-optimization model
Romano, Guizzi, and Chiocca [116]	System dynamics simulation, Lean techniques, Causal loop diagram
Ross et al. [118]	Multivariate logistic regression, Ordinary least squares regression
Ryan et al. [218]	Lean manufacturing, Theory of constraints, Logistic regression
Shirazi [219]	Simulation-based optimization
Stanton et al. [220]	Lean Six Sigma
Weimann [221]	Standardized project management, Change management, Continuous quality improvement, Lean manufacturing
Yousefi and Ferreira [125]	Agent-based simulation, Group Decision Making
Zeinali, Mahootchi, and Sepehri [184]	Discrete-event simulation, Metamodels, Cross validation

**Table 6:** Articles evidencing the use of process improvement techniques for reducing LWBS.

Authors	Technique Type
Single	
Carter et al. [50]; Dickson et al. [52]; Kane et al. [56]; Murrell, Offerman, and Kauffman [58]; Ng et al. [59]; Peng, Rasid, and Salim [60]; Sánchez et al. [63]; Sayed et al. [64]; Van der linden et al. [65]; Vashi et al. [149]; Vermeulen et al. [66]	Lean manufacturing (S)
Preyde, Crawford, and Mullins [81]; Rehmani and Amatullah [82]; Rothwell, McIltrot, and Khouri-Stevens [155]; Welch and Allen [224]	Continuous quality improvement (S)
Chan et al. [91]	Rapid Entry and Accelerated Care at Triage (REACT)
Christensen et al. [92]	Pivot nursing
Schwab et al. [225]	Statistical Process Control
DeFlitch et al. [94]	Process redesign
Hybrid	
Easter et al. [25]	Discrete-event simulation, ANOVA, Linear regression, Non-linear regression
Hitti et al. [222]	Logistic regression, Case-control study
Jiang, Chin, and Tsui [223]	Deep neural network (DNN), Genetic algorithm (GA)
Lee et al. [112]	Machine learning, Simulation, Optimization
Yousefi and Ferreira [125]	Agent-based simulation, Group Decision Making
Yousefi et al. [127]	Agent-based simulation, Ordinary least squares regression
